# Adipose Tissue Plasticity: A Comprehensive Definition and Multidimensional Insight

**DOI:** 10.3390/biom14101223

**Published:** 2024-09-27

**Authors:** Yu-Yao Mo, Yu-Xin Han, Shi-Na Xu, Hong-Li Jiang, Hui-Xuan Wu, Jun-Min Cai, Long Li, Yan-Hong Bu, Fen Xiao, Han-Dan Liang, Ying Wen, Yu-Ze Liu, Yu-Long Yin, Hou-De Zhou

**Affiliations:** 1 National Clinical Research Center for Metabolic Diseases, Hunan Provincial Key Laboratory for Metabolic Bone Diseases, Department of Metabolism and Endocrinology, The Second Xiangya Hospital of Central South University, Changsha 410011, China; moyuyao@csu.edu.cn (Y.-Y.M.); 228201009@csu.edu.cn (Y.-X.H.); 248201016@csu.edu.cn (S.-N.X.); hollyy@csu.edu.cn (H.-L.J.); susywu2010@gmail.com (H.-X.W.); jmcai2021@csu.edu.cn (J.-M.C.); cogebe@csu.edu.cn (L.L.); 218201015@csu.edu.cn (F.X.); 238207001@csu.edu.cn (H.-D.L.); 228211055@csu.edu.cn (Y.W.); 2 Department of Blood Transfusion, The Second Xiangya Hospital, Central South University, Changsha 410012, China; 504475@csu.edu.cn; 3 Pediatric Cardiac Surgery Centre, Fuwai Hospital, National Centre for Cardiovascular Diseases, State Key Laboratory of Cardiovascular Disease, Chinese Academy of Medical Sciences, Peking Union Medical College, Beijing 100730, China; liuyuze@fuwai.com; 4 Institute of Subtropical Agriculture, Chinese Academy of Sciences, Changsha 410125, China

**Keywords:** adipose tissue plasticity, adipocytes, obesity, therapeutic strategy

## Abstract

Adipose tissue is composed of adipocytes, stromal vascular fraction, nerves, surrounding immune cells, and the extracellular matrix. Under various physiological or pathological conditions, adipose tissue shifts cellular composition, lipid storage, and organelle dynamics to respond to the stress; this remodeling is called “adipose tissue plasticity”. Adipose tissue plasticity includes changes in the size, species, number, lipid storage capacity, and differentiation function of adipocytes, as well as alterations in the distribution and cellular composition of adipose tissue. This plasticity has a major role in growth, obesity, organismal protection, and internal environmental homeostasis. Moreover, certain thresholds exist for this plasticity with significant individualized differences. Here, we comprehensively elaborate on the specific connotation of adipose tissue plasticity and the relationship between this plasticity and the development of many diseases. Meanwhile, we summarize possible strategies for treating obesity in response to adipose tissue plasticity, intending to provide new insights into the dynamic changes in adipose tissue and contribute new ideas to relevant clinical problems.

## 1. Introduction

Adipose tissue (AT) is a highly active, flexible organ with great remodeling ability and functional pleiotropism [1], and its significance in systemic physiology has recently been highlighted [2]. Because AT includes mature adipocytes, blood vessels, neurons, fibroblasts, and different immune cells such as macrophages, the extracellular matrix (ECM), adipose stem cells (ASCs), and preadipocytes, it is considered a tissue [3]. These many elements work in sync in AT to sustain AT homeostasis. AT is divided into white and brown ATs (WAT and BAT, respectively), and beige AT is the portion of WAT that resembles BAT. WAT and BAT have unique functions and are closely related during the body’s adaptation to changes in energy demand. WAT, including subcutaneous AT (SAT) and visceral AT (VAT), stores energy in the form of lipid droplets. These droplets protect the remaining body’s organs from the toxicity of ectopic lipid deposits [4]. Thermogenic AT (brown and beige) generates heat by oxidizing fatty acids, etc., thereby facilitating energy expenditure and obesity prevention [5]. The majority of thermogenic adipocyte heat production depends on the uncoupling protein 1 (UCP1) pathway in the inner mitochondrial membrane, and free fatty acids (FFAs), glucose, lactate, and ketone bodies act as thermogenic fuels [6,7].

Excessive fat storage leads to individuals being affected by overweight or obesity. This results in a series of health disorders, including metabolic syndrome, stroke, coronary heart disease, and type 2 diabetes mellitus (T2DM) [8]. The global prevalence of obesity is continuously increasing. At present, approximately one-third and one-tenth of the global population are affected by overweight and obesity, respectively [9].

Recent advancements in technology such as single-cell RNA sequencing (scRNA-seq) and single-nucleus sequencing (snRNA-seq) have facilitated our understanding of the heterogeneity and functionality of AT depots [10,11,12]. We herein present a new and complete definition of AT plasticity for the first time. According to us, AT plasticity is defined as the active adaptability of AT to undergo significant changes in its various tissue components under physiological or pathological conditions up to a certain threshold. These changes include those in adipocyte size and number, blood vessels and nerves, infiltrating immune cells, adipokine secretion, differentiation and dedifferentiation capacity, etc. We also highlight the multifaceted interpretation of AT plasticity and the presence of certain thresholds as well as individual variability for this plasticity. We also elaborate on possible strategies related to obesity treatment.

## 2. Plasticity in Adipocyte Size

**Figure 1:**
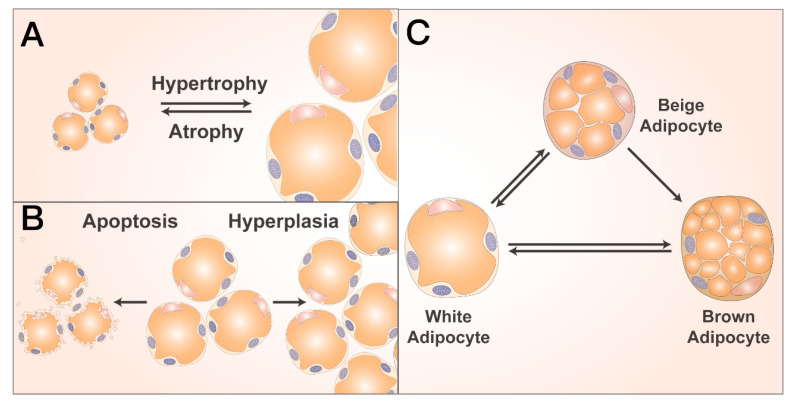
The plasticity in adipocyte size, number, and species. (**A**) Plasticity in the adipocyte size. Adipocyte size plasticity is accomplished by hypertrophy or atrophy. (**B**) Plasticity in the adipocyte number. The increase in the number of adipocytes occurs through cell proliferation, while the decrease in the number of adipocytes is promoted by apoptosis. (**C**) Plasticity in adipocyte species. The white adipose tissues and brown adipose tissues can be interconverted under certain conditions (such as browning and whitening).

Adipocytes can enlarge or contract to adjust to regional and systemic metabolic changes (Figure 1A). This size change is accomplished by adipocyte hypertrophy or atrophy [13]. Adipocytes are highly plastic, and their size can increase from 20 μm to hundreds of micrometers in diameter during hypertrophy, and this mainly depends on the triglyceride content [14]. Adipocyte hypertrophy increases inflammation and cell death [15], and excessive adipocyte hypertrophy may be a stress that causes ongoing inflammation in AT [16]. The hypertrophy of subcutaneous abdominal adipocytes is a hallmark of increased WAT in people with obesity, which is positively associated with glucose tolerance and hyperinsulinemia and may increase T2DM risk [17].

Adipocyte size is regulated by various environmental and physiological factors. Prolonged exposure to mild cold enlarges BAT mass and activity, which is restored when AT is exposed to a warm environment [18]. Research has demonstrated a noticeable increase in visceral fat, particularly in the epididymal WAT, in mice with advancing age. Morphological investigations have corroborated that this augmentation primarily arises from the enlargement of adipocytes. Certain dietary interventions, such as a 72 h fasting regimen, have been observed to induce a significant reduction in adipocyte size among mice, with more pronounced effects observed in younger cohorts. Following a 72 h refeeding period, adipocytes in younger mice reverted to their initial dimensions, whereas those in adult and senescent mice exhibited sustained but marginally reduced sizes compared to pre-fasting levels [19]. An organism’s sex and AT location also seem to regulate adipocyte size plasticity. Females have smaller adipocytes in the omentum than in the SAT, whereas males typically have larger adipocytes and have little difference in adipocyte size in both sites [20]. This might be related to sex hormones, as the aforementioned differences in AT morphology decrease in women after menopause, more closely resembling the expression pattern in men [21].

In vivo, adipocyte hypertrophy and its degree are also regulated by local and systemic factors. As adipocyte size increases, the adipocytes come in contact with adjacent cells and ECM components and are subjected to increased mechanical stress and decreased oxygen diffusion. This leads to hypoxia and AT inflammation at the threshold, thus preventing the increase in adipocyte size [22]. Adipocyte size is suggested to be regulated by volume-sensitive adipocyte membrane proteins, such as SWELL11 [23]. The expression of inflammation-related genes NF-κB and tumor necrosis factor (TNF)-γ is positively correlated with adipocyte size. This reflects that increased adipocyte size is correlated with increased inflammatory responses [24]. The expression of immune-related genes such as serum amyloid A is also significantly higher in large adipocytes [25]. Adipokine secretion also has a role in size regulation, with extra-large adipocytes (>127 μm) exhibiting an increased secretion of pro-inflammatory adipocytokines interleukin-6 (IL-6), monocyte chemotactic protein-1 (MCP-1), and IL-8 compared with small adipocytes (<73 μm) [15], Increased expression of lipid droplet surface proteins Cell death induces DFFA-like effector protein A (CIDEA) and Fat-specific Protein 27 in the SAT may increase adipocyte size by increasing triglyceride storage [26]. The metabolic rhythms of peroxisome proliferator-activated receptor γ (PPARγ) acetylation also influences adipocyte size [27].

## 3. Plasticity in Adipocyte Number

To increase or decrease adipocyte number, new cells must be produced de novo, and existing cells must be destroyed (Figure 1B) [13]. Although most adipose growth occurs during childhood and adolescence, with fluctuations in energy balance, WAT can continue to undergo expansion during adulthood [28].

The increase in adipocyte number occurs mainly through cell proliferation, often also called adipogenesis, involving the transition from multipotent mesenchymal stem cells (MSCs) to preadipocytes and then to adipocytes [29]. The specific molecular regulatory mechanisms are mentioned subsequently (8.1). As confirmed in many experiments, AT expansion occurs primarily from adipocyte proliferation, thereby preventing metabolic diseases by maintaining the function of adipocytes and adequate lipid storage capacity within them. Lu et al. induced proliferative SAT expansion by injecting an adipose cocktail in mice, thereby demonstrating improved glucose tolerance and insulin sensitivity [30]. PPARγ agonist thiazolidinediones (TZD) improve systemic insulin sensitivity through proliferative SAT expansion despite weight gain [31].

Adipocyte hyperplasia rather than adipocyte hypertrophy manifests increased VAT mass in the population affected by obesity [32]. The proliferative capacity of VAT is greater than that of SAT. Although adipocyte proliferation in SAT increases during diet-induced obesity, adipogenesis may be limited to VAT at obesity onset [33].

Adipocyte numbers may decrease through apoptosis. Adipocyte apoptosis occurs under physiological conditions, with increased apoptotic marker expression in mature adipocytes in a thermoneutral (30 °C) environment compared with controls (22 °C) [34]. Untargeted metabolomics have revealed that apoptotic brown adipocytes release highly enriched purine metabolites, thereby enhancing the expression of thermogenic programs in healthy adipocytes [35]. Adenine nucleotide translocase 2 (ANT2) participates in adipocyte apoptosis, and ANT2 expression is downregulated during cold stimulation with a corresponding decrease in apoptosis ([36], p. 2). In people with morbid obesity, apoptosis increases with the exacerbation of metabolic-associated fatty liver disease (MAFLD) [37].

There is a strong link between changes in adipocyte size and their overall number. In SAT, the total number of adipocytes tends to decrease as their size increases, and fewer new adipocytes are formed in hypertrophic AT [38].

## 4. Plasticity of Adipocyte Species

### 4.1. Browning

Browning is defined as the transformation of WAT into tissue with BAT-like function and activity. It involves changes in the lipid droplet structure, robust mitochondrial biogenesis, and the upregulation of transcriptional programs supporting high levels of local fuel oxidation (Figure 1C) [39]. In healthy physiological conditions, BAT plays a crucial role in thermogenesis and energy expenditure, whereas WAT primarily functions as an energy reservoir. However, during obesity, WAT can become dysfunctional due to chronic inflammation, leading to impaired metabolic regulation. This dysfunction increases the risk of cardiovascular disease and T2DM. WAT browning is a potential therapeutic approach, as BAT activation prevents the deleterious effects of dysfunctional white adipocyte-induced lipid overflow [33,40].

#### 4.1.1. Transcriptional and Epigenetic Regulation

Several proteins, including PPARγ coactivator 1α (PGC-1α) [41], PR-domain-containing 16 (PRDM16) [42], and early B-cell factor 2 [43], interact with PPARγ to regulate WAT browning. Many members of the bone morphogenetic protein (BMP) family, a subgroup of the transforming growth factor-β (TGF-β) superfamily, are involved in regulating browning. BMP4 promotes browning and improves insulin sensitivity [44], and BMP7 functions in WAT browning by increasing the expression of the transcriptional regulators PRDM16, PGC-1α, and PPARγ [45,46]. Experiments have demonstrated that the knockdown of SMAD3, which is part of the TGF-β signaling pathway, induces AT browning and prevents obesity and diabetes. The deletion of the transcription factor FoxO1 can lead to the same outcome [47]. This suggests that TGF-β is involved in browning [48]. Genetically inhibited Notch signaling may also promote WAT browning and inhibit obesity [49]. Many epigenetic regulatory molecules such as histone acetyltransferases, histone deacetylases, histone methyltransferases, and histone demethylases are associated with WAT browning [50]. miRNAs play a bidirectional role in the activation of BAT and beige adipose tissue [51]. Regarding positive regulation, under cold conditions, miR-455 is specifically expressed in BAT to activate brown adipocytes and adipose-specific miR-455 transgenic mice exhibited browning in the SAT [52]. miR-199a/214 [53], miR-155 [54], miR-327 [55], miR-19b [56], miR-133 [57], and miR-34a [58] are key negative regulators that inhibit the WAT-to-BAT conversion and beige adipocyte recruitment.

#### 4.1.2. Endocrine Hormone Regulation

Catecholamines are browning-inducing hormones that act through β-adrenergic receptors (βARs) on adipocytes to promote browning and increase thermogenesis [59]. Under cold conditions, the sympathetic nervous system is activated and norepinephrine (NE) release increases, thereby binding and activating the βARs on adipocytes. βARs then interact with the GTP-binding protein GS to activate adenylate cyclase, thereby increasing cellular cAMP levels and activating cAMP-dependent protein kinase A (PKA). PKA phosphorylates and modifies lipid droplet-binding proteins A and B and some lipases, such as adipose triglyceride lipase and hormone-sensitive triglyceride lipase, to promote FFA and glycerol release [60,61]. The released FFA activates the UCP1 protein and acts as a substrate for UCP1-mediated thermogenesis [62]. The p38 pathway also has a regulatory role in the aforementioned thermogenesis [63]. Curcumin enhances the expression of β3-adrenergic receptor genes in SAT in mice, thereby increasing plasma NE levels and enhancing thermogenesis [64]. Of note, even in the absence of βARs, cold stimulation can induce beige adipocyte production to promote thermogenesis. This particular beige adipose tissue is known as glycolytic beige (g-beige) adipose tissue [65]. Thyroid hormones (THs) also induce WAT browning and activate BAT function. Serum T4 levels are positively correlated with UCP1, CIDEA, and PRDM16 in WAT [66]. The thyrotropin receptor (TR) agonist-mediated induction of browning may be a treatment-appropriate strategy for obesity, and treatment with the TR agonist GC-1 promotes WAT browning and reduces obesity and diabetes in mice [67]. Coordinated synergistic effects between glucagon and thyroid hormones may correct glucose tolerance and obesity [68].

#### 4.1.3. Environmental and Lifestyle Effects

During exercise, skeletal muscle contraction releases a fibronectin-like peptide called irisin. This hormone, produced in both mice and humans, upregulates PPARα and UCP1 expression, thereby acquiring the ability to induce WAT browning [69], positively affect energy metabolism [70], and improve high-fat diet (HFD)-induced insulin resistance (IR) [71]. The brain-derived neurotrophic factor (BDNF), also released after exercise, induces WAT browning and increases energy expenditure [72]. Lactate production during anaerobic exercise may also lead to WAT browning [73].

The levels of circulating fibroblast growth factor 21 (FGF21) are elevated under cold conditions. FGF21 may promote WAT browning through FGFR1 and scaffold βKlotho, thereby promoting thermogenesis [74,75]. FGF15/19, FGF family members, are also associated with AT plasticity during thermogenic adaptation. FGF19 levels in humans directly correlate with *UCP1* expression in the SAT, whereas increased FGF15 or FGF19 levels in mice induce WAT browning [76]. Cold-activated BAT leads to an enhanced uptake of triglyceride-rich lipoproteins through lipoprotein lipase and the transmembrane receptor CD36, thereby accelerating serum triglyceride removal [77]. Although burns also cause WAT browning, this browning is impaired in old patients and is associated with reduced survival in old burn patients [78].

Intermittent fasting selectively stimulates beige adipocyte development in WAT and reduces obesity by modulating the gut microbial composition in order to increase acetate and lactate biosynthesis [79]; acetate [80] and lactate [81] have been proven to be browning agents.

### 4.2. Whitening

When various external stimuli are removed, such as reheating adipocytes after cold stimulation, catecholamine stimulation is no longer present, and beige or brown adipocytes tend to lose their characteristics. Moreover, they exhibit an increase in white adipocyte characteristics. This process is known as “whitening.” The modifications include a change in internal multilocular lipid droplets to unilocular forms, reduction in mitochondria, and decreased UCP1 expression (Figure 1C). [82]. Although the identity of beige adipocytes is altered, their epigenomic memory is retained from previous cold exposure, and hence, they can activate thermogenic genes faster when the next cold stimulus is applied, which is associated with the action of poised enhancers. This particular process is not evident in brown adipocytes [83]. The glucocorticoid receptor (GR) and its downstream target Zfp423 have a crucial role in whitening. Inguinal subcutaneous WAT (iWAT) from both adipocyte-specific GR knockout mice and adipocyte-specific Zfp423 knockout mice have exhibited delayed whitening after rewarming [83].

## 5. Plasticity of the Internal Structure of Adipocytes

Internally, adipocytes include lipid droplets, mitochondria, and the nucleus. Under different physiological or pathological conditions, the size and number of lipid droplets as well as the number and activity of mitochondria in adipocytes change significantly to adapt to the energy demand at different stages. Lipid storage in adipose tissue involves the uptake of circulating fatty acids and glucose, which are converted into triglycerides and stored in lipid droplets within adipocytes. Conversely, lipolysis is the process by which these stored triglycerides are broken down into free fatty acids and glycerol, which are released into the bloodstream for energy use. These processes are tightly regulated by hormonal signals, with insulin promoting lipid storage and catecholamines stimulating lipolysis [84]. The expression level of UCP1 protein, a part of the mitochondrial inner membrane associated with heat production, also fluctuates under the aforementioned conditions. For the WAT-to-BAT or WAT-to-beige AT transition, a reduction in adipocyte size and an increase in mitochondrial content is necessary, and this moderate browning serves to protect WAT [85]. Specifically, WAT shows unilocular large lipid droplets, whereas BAT and beige AT exhibit multilocular small lipid droplets. Mitochondrial content and UCP1 levels gradually increase in the order of white to beige to brown adipocytes [86]. Plasticity has also been observed in mitochondrial activity, with atrial natriuretic peptide (ANP) and brain natriuretic peptide (BNP) released by cardiomyocytes during cold exposure. ANP enhances BAT mitochondrial activity in a p38 mitogen-activated protein kinase (p38 MAPK)-dependent manner [87], while BNP increases mitochondrial biosynthesis in WAT [88]. Multiple factors influence these changes. Rosiglitazone, a PPARγ agonist, enhances the expression of UCP1 and mitochondrial thermogenic genes in 3T3-L1 adipocytes [89]. THs and the sympathetic nervous system interact to increase UCP1 expression in BAT [90]. Autophagy is also involved in intracellular remodeling events occurring during brown/beige adipogenesis, and thermogenic activation and inactivation [91].

## 6. Plasticity of the External Matrix of Adipocytes

Along with adipocytes, AT contains many other cell types and structures, including adipose endothelial cells (AdECs) associated with tissue vascularization, nerve endings, non-differentiated precursor cells in various stages, and immune cells [16]. These cells and structures also have some degree of plasticity.

### 6.1. Plasticity of Blood Vessels in Adipose Tissue

**Figure 2:**
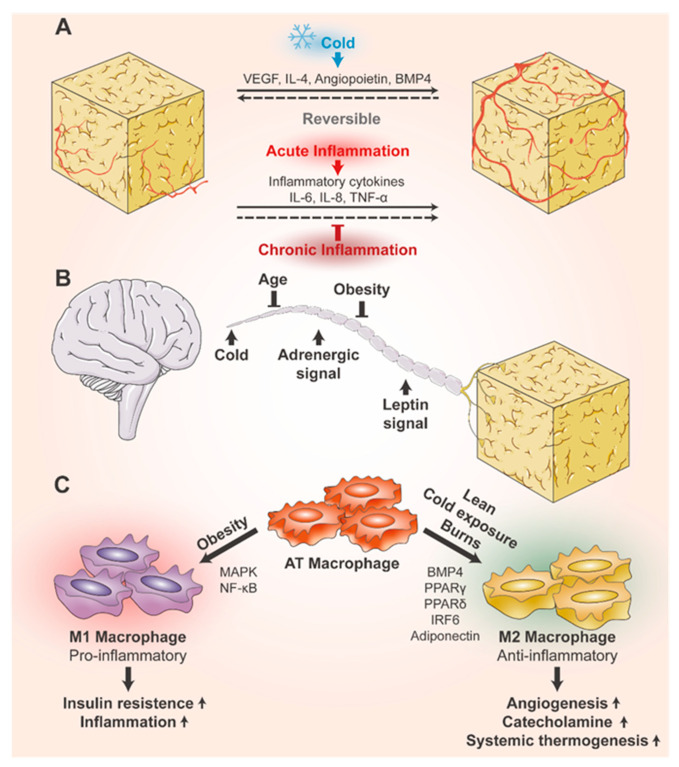
The plasticity in the external matrix of adipocytes. (**A**) Plasticity of blood vessels in adipose tissue. Under cold conditions, adipose tissue angiogenesis is induced and regulated by factors such as vascular endothelial growth factor (VEGF), interleukin-4 (IL-4), angiopoietin, and bone morphogenetic protein 4 (BMP4). This process is reversible upon the removal of the cold stress. During acute inflammation, adipose tissue angiogenesis is promoted by factors such as interleukin-6 (IL-6), interleukin-8 (IL-8), and tumor necrosis factor-α (TNF-α). However, persistent chronic inflammation inhibits this process due to prolonged hypoxia. (**B**) Plasticity of nerves in adipose tissue. The cold exposure, the activation of adrenergic, and leptin signaling lead to an increased density of sympathetic axons in adipose tissue, facilitating enhanced thermogenesis. In contrast, this adaptive process is impeded in pathological conditions such as aging or obesity. (**C**) Plasticity of immune cells in adipose tissue. In obesity, there is an augmented conversion of adipose tissue macrophages into pro-inflammatory M1 macrophages, fostering the development of insulin resistance and inflammation. Conversely, in lean populations or under conditions such as cold exposure or burns, there is an increased transformation of adipose tissue into anti-inflammatory M2 macrophages, promoting angiogenesis, catecholamine production, and enhanced thermogenesis. The activation of MAPK signaling and NF-κB signaling pathways may be associated with M1 polarization, while BMP4, PPAR γ, PPAR δ, IRF-6, and adiponectin contribute to M2 polarization.

Inflammation, fibrosis, and impaired angiogenesis are the triad of adipose tissue dysfunction. The relationship between these three conditions is complex, and the relationship between inflammation and angiogenesis in AT remains unclear [92]. AT function and growth are dependent on angiogenesis within the tissue. Blood flow supplies oxygen to the AT and transports nutrients, waste products, and various hormones and cytokines [93]. Various signaling molecules in the AT microenvironment are involved in the modulation of the adipose vascular system (Figure 2A). In healthy physiological conditions, such as exposure to cold, angiogenesis is positively regulated. This process is driven by the production of angiogenic factors, including vascular endothelial growth factor (VEGF), IL-4, and angiopoietin, which induce new blood vessel formation to meet the increased metabolic demand of the tissue [94]. Such changes are reversible, and recovery is possible after stress removal [39]. BMP4, as an angiogenic molecule, may also act as an essential regulator of this process [95]. On the other hand, in pathological conditions, the regulation of angiogenesis becomes more complex. but when this pathway is impaired—such as through PPARγ silencing—AdEC angiogenic activity decreases, leading to reduced FFA uptake and exacerbating inflammation [96]. In acute inflammation, angiogenesis can be positively regulated by inflammatory cytokines like IL-6, IL-8, and TNF-α, which promote the formation of new blood vessels to support tissue repair [97]. However, during chronic inflammation, often triggered by sustained AT expansion and hypoxia, angiogenesis becomes impaired, contributing to tissue dysfunction [92]. Interestingly, anti-inflammatory M2 macrophages are more pro-angiogenic compared with pro-inflammatory M1 macrophages [98]. ASCs in AT may be involved in angiogenesis by secreting miRNA-containing exosomes, such as miR126, miR31, miR92a, miR221, miR30, miR100, and miR486, which can directly regulate angiogenesis [94]. The latest 3D volume fluorescence-imaging procedure reveals that catecholamine signaling exerts crucial effects on AT vascular remodeling. The genetic deletion of the three βAR types would completely inhibit cold-induced vascular plasticity in WAT [99].

### 6.2. Plasticity of Nerves in Adipose Tissue

Sympathetic nerves in AT regulate the thermogenic capacity of adipocytes, and sympathetic nerve density varies with energy balance requirements [100]. Sympathetic nerves are the route of communication from the brain to AT that facilitates the regulation of adipocyte number and size as well as various functional changes [101] (Figure 2B). The plasticity of AT nerves depends on nerve growth factor (NGF)- and TrkA receptor-mediated cold-evoked signaling. When NGF or TrkA signaling is blocked, sympathetic nerve plasticity in AT is inhibited, thereby preventing the conversion of WAT to beige adipocytes under cold stimulation [102]. Eosinophils can also modulate sympathetic plasticity. Sympathetic activation leads to IL-33 release from stromal cells through adrenergic signaling, which then acts on innate lymphoid cells2 and induces IL-5 production. IL-5 is essential for adipose eosinophil regulation, and eosinophils subsequently regulate axonal growth within the adipose [100]. For the maintenance of sympathetic structural plasticity, leptin signaling is critical. Mice with leptin gene mutations (ob/ob mice) exhibit a significant reduction in sympathetic innervation in subcutaneous WAT and BAT, which is restored after chronic leptin treatment [103]. Leptin exerts its effects on innervation through the agouti-related peptide and pro-opiomelanocortin neurons in the arcuate nucleus of the hypothalamus, acting through brain-derived neurotropic factor-expressing neurons in the paraventricular nucleus of the hypothalamus (BDNF^PVH^) [103]. Cold-induced neuroimmune cells (CINCs) of bone marrow lineage origin that also secrete BDNF have a role in promoting neural remodeling in iWAT, where CINCs are increasingly recruited to AT and promote axonal survival during cold stimulation [101]. With advancements in techniques such as adipose tissue innervation imaging, sensory nerves have also been shown to play a role in regulating lipolysis, promoting angiogenesis, and in interacting with sympathetic nerves [104]. Under some pathological conditions, including aging and obesity, WAT fails to maintain proper innervation and undergoes “adipose neuropathy”, exhibiting reduced protrusions, loss of synaptic markers, and reduced local expression of neurotrophic factors [105].

### 6.3. Plasticity of Immune Cells in Adipose Tissue

The scattered distribution of numerous immune cells in AT is closely related to the development of inflammation and obesity-related metabolic disorders [106]. The plasticity of immune cells infiltrating AT is also significant, mainly in the composition of macrophage subsets. Most AT macrophages (ATMs) infiltrating obese fat are arranged around the remnants of dead adipocytes, forming distinctive “crown-like structures” [85]. In obesity, lipid-associated macrophages (LAMs) and proliferative LAMs are significantly increased and become the “dominant” macrophage subpopulation. The expression of cytokines such as chemokine ligand 8, osteopontin, and progranulin is dramatically upregulated in obese macrophages [107]. Macrophages tend to act as pro-inflammatory M1 macrophages in most populations with obesity, whereas they predominantly act as anti-inflammatory M2 macrophages in lean populations [108] (Figure 2C). The activation of MAPK signaling [109] and NF-κB signaling [110] pathways may be associated with ATM polarization to M1 macrophages. BMP4 activates M2 macrophages and inhibits M1 macrophage polarization, and activated M2 macrophages also promote AT angiogenesis [111]. PPARγ [112], PPARδ [113], interferon-regulatory factor-6 [114], and adiponectin [115] are also believed to contribute to M2 polarization. In the WAT of people affected by obesity, M1 macrophages are increased, resulting in pro-IR and pro-inflammatory conditions. Although the absolute number of M2 macrophages increases with obesity development, they continue to be less numerous than M1 macrophages in relative terms [116]. Interestingly, cold exposure or burns are associated with macrophage infiltration, with an increase in M2 macrophages in adipose tissue in both cases. Catecholamine production was promoted, promoting WAT browning and increasing systemic thermogenesis [117,118]. Exercise and cold exposure-induced circulating factor meteorin-like (Metrnl) also promote alternative macrophage activation in AT, correlating with the expression of anti-inflammatory genes in AT [119]. Unlike macrophages in other tissues, macrophages in AT also regulate lipid metabolism and recycle lipids from adipocytes as well as catecholamines from neurons [120].

In addition to that of macrophages, B-2 and T cell plasticity in AT has been reported. B-2 cells accumulate in the AT of diet-induced obese mice, and increased IgG2c antibodies derived from B-2 cells mediate IR [121]. Cytotoxic T cells (CD8+ T cells) are increased in obesity-induced inflammation at the early stages and are related to macrophage recruitment and activation [122]. CD4+ helper T cell 1 (Th1) promotes inflammation in obesity development and increases in number, while CD4+ helper T cell 2 (Th2) has an anti-inflammatory role and decreases in number [123]. VAT Foxp3 regulatory T cells (Tregs) protect AT from inflammation during obesity, a process in which PPAR-γ expression is crucial [124]. The beneficial effects of VAT Tregs and M2 macrophages can be maintained through the synergistic effect of invariant natural killer T cells, which are associated with IL-2 and IL-10 secretion [125].

## 7. Plasticity of Adipose Tissue Distribution

**Figure 3:**
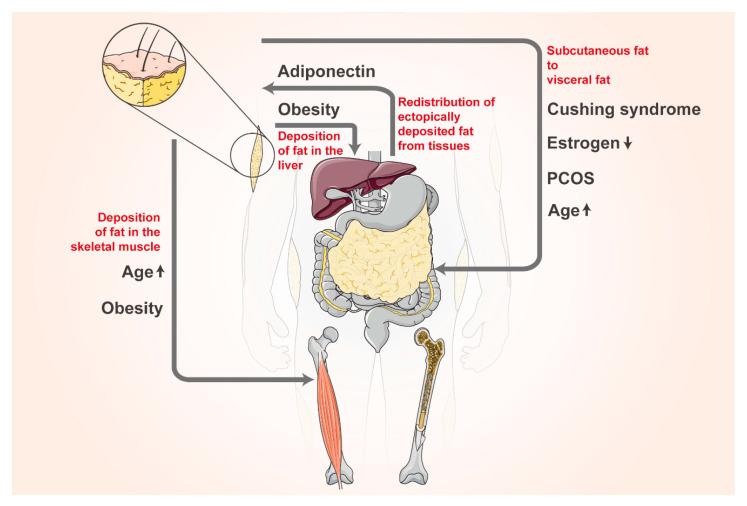
Plasticity of adipose tissue distribution. Pathological conditions such as Cushing’s syndrome and polycystic ovary syndrome (PCOS), along with physiological changes like menopause and aging, contribute to the shifting of subcutaneous fat to visceral fat. With aging and obesity, there is also an observed ectopic deposition of fat in the liver and skeletal muscle. Notably, the role of adiponectin is paramount in promoting the redistribution of ectopically deposited fat from tissues, such as the liver, back to the subcutaneous fat depot.

AT distribution in humans can be classified into SAT, VAT, and bone marrow fat. SAT primarily serves as an energy reservoir and is associated with lower health risks, while VAT is linked to metabolic disorders, including insulin resistance and cardiovascular disease [126]. AT distribution can also shift under some physiological or pathological conditions or the effect of hormones or drugs (Figure 3). For instance, in patients with Cushing’s syndrome, glucocorticoids cause a greater migration of systemic lipids to visceral fat and increased fat consumption in the extremities [127]. AT redistribution from the periphery of the body to the abdomen under physiological conditions seems to result from aging and the effect of sex hormones, thereby adapting to a lower basal metabolic rate and reduced physical activity [128]. Hormones impact the plasticity of AT distribution; for example, postmenopausal women often experience an increase in VAT due to declining estrogen levels, whereas estrogen supplementation may help reduce this effect [129]. Similarly, hyperandrogenemia in patients with polycystic ovary syndrome (PCOS) also stimulates greater fat accumulation toward visceral adiposity [130].

Aging also causes changes in fat distribution, and most of the population aged >65 year tend to accumulate fat as the visceral fat depot, resulting in visceral obesity [131]. Adiponectin releases the “adipocyte starvation” signal to the body, facilitating the transfer of lipids from ectopic sites, such as the liver and muscle, to SAT, which helps lower blood lipid concentrations [132]. Additionally, the ethanolic extract of the *Platycodon grandiflorus* root also reduces blood lipid levels by increasing FFA uptake in epididymal WAT [133].

In a population affected by obesity, fat deposition also occurs in some non-adipose tissues such as the liver and skeletal muscle. This ectopic fat accumulation is often linked to the age-related dysregulation of lipid metabolism in SAT [134]. Lipotoxicity refers to the harmful effects of excess lipids on non-adipose tissues, which can disrupt cellular function and lead to metabolic disorders. However, the presence of adipokines, such as adiponectin, can mitigate lipotoxicity by facilitating the transfer of excess lipids from these ectopic sites back to adipocytes, thereby reducing the burden on non-adipose tissues and improving overall metabolic health in individuals with obesity [132].

## 8. Plasticity of Adipocyte Differentiation and Dedifferentiation Capacity

### 8.1. Plasticity of Adipocyte Differentiation Capacity

BAT originates in the paraxial mesoderm, while WAT originates in the mesoderm and neural crest. The different origins of BAT and WAT may influence their different plasticity. Adipocyte differentiation can be understood as adipocyte generation. The first step in AT generation is the spectrum commitment of MSCs to preadipocytes, followed by the growth arrest and differentiation of preadipocytes into lipid-containing functional mature adipocytes [135]. MSCs are precursors of adipocytes as well as common progenitors of myogenic, osteogenic, and chondrogenic cells [136]. Adipose-derived stem cells (ADSCs) exhibit great plasticity and are a potential driving force for stem cell therapy because of their relative accessibility and extensive self-renewal potential [137]. Increasing ADSC conversion to osteoblasts, transplanting autologous osteogenic pre-differentiated ADSCs into the fracture gap, and forming seed cells for bone tissue engineering may be one of the possible therapeutic avenues for refractory fractures [138]. PPARγ [139] and the coactivator CCAAT/enhancer binding protein α (C/EBPα) are the main regulators of adipogenesis [140]. However, none of these regulators are highly expressed in early adipogenesis. Instead, their transcriptional activator C/EBPβ shows transiently high expression, implying that it may act as a key regulator of early adipogenesis [141]. Interestingly, a study found that PPARγ phosphorylation correlates with sex differences in the differentiation of iWAT adipocyte precursor cells (APCs). Namely, in male HFD-fed mice, iWAT APCs were resistant to adipogenesis, whereas perigonadal WAT (gWAT) APCs were activated and pro-adipogenic. By contrast, in HFD-fed female mice, de novo adipogenesis was observed in both iWAT and gWAT [142]. Krüppel-like factors (Klfs), transcriptional regulators, are also highly expressed during early adipogenesis. Among them, Klf4, Klf5, and Klf15 are positively involved in adipogenic differentiation [143], and Klf4 can bind to the C/EBPβ promoter to promote PPARγ and Klf15 expression ([144], p. 4). By contrast, Klf2, Klf3, Klf7, and Klf9 are negatively associated with adipogenic differentiation [143]. TGF-β is also a member of adipogenesis suppressors. These suppressors can inhibit adipogenesis by activating SMAD proteins to promote the expression of ECM-related genes such as collagen genes [145]. Some BMP members in the TGF-β superfamily perform different functions. BMP2 and BMP4 can effectively promote adipocyte production through the BMP-SMAD-lysyl oxidase pathway [146], while BMP7 can promote brown adipocyte production by increasing PRDM16 and PGC-1α [45]. Matrix metalloproteinase 11 (also known as globular plasmolysin 3) in the AT microenvironment acts as a negative regulator of adipogenesis [147].

### 8.2. Plasticity of Adipocyte Dedifferentiation Capacity

Dedifferentiation, a mechanism of adipocyte plasticity, is the conversion of mature adipocytes back to an undifferentiated progenitor cell-like state [13]. Mature adipocytes successfully cultured using the “ceiling culture” method can, under certain conditions, lose their lipid droplets and revert to progenitor-like cells, called dedifferentiated adipocytes. Dedifferentiated adipocytes have a multispectral differentiation potential both in vivo and in vitro and expresses various ASC markers, suggesting stem cell properties [148]. Under physiological conditions, such as during lactation, adipocytes may lose lipid droplets and dedifferentiate into fibroblast-like cells [13], whereas, during wound healing, myofibroblasts regenerate and differentiate into adipocytes, implying that dedifferentiation is reversible [149]. The dedifferentiation ability of adipocytes is also reflected in tumor progression. In breast cancer, adipocytes present around cancer cells transform into fibroblast-like cells and express high levels of cancer cell markers, thereby driving breast cancer progression [150]. Aberrant Notch signaling activation drives adipocyte dedifferentiation and the transformation of these adipocytes into liposarcoma cells [151]. When 3T3-L1 adipocytes and the pancreatic cancer cells MiaPaCa2 are co-cultured in vitro, MiaPaCa2 cells release Wnt5a, which target Wnt signaling in 3T3-L1 adipocytes. Treatment with secreted trichome-associated protein 5, a Wnt5a inhibitor, blocks downstream Wnt signaling in 3T3-L1 cells and prevents their dedifferentiation [152]. Bleomycin promotes the transformation of adipocytes to myofibroblasts in the SAT [153] and can inhibit adipogenesis by enhancing TGF-β1 expression ([154], p. 1). Adipocyte dedifferentiation is potentially valuable for applications, as inhibiting peritumor adipocyte dedifferentiation or promoting fibroblast-like cell redifferentiation in breast cancer may inhibit breast cancer progression. The reversibility of adipocyte dedifferentiation also suggests a broad range of applications for DFAT cells, which may be used in wound healing.

## 9. Plasticity of Adipocytes Secreting Adipokines or Extracellular Vesicles

**Figure 4:**
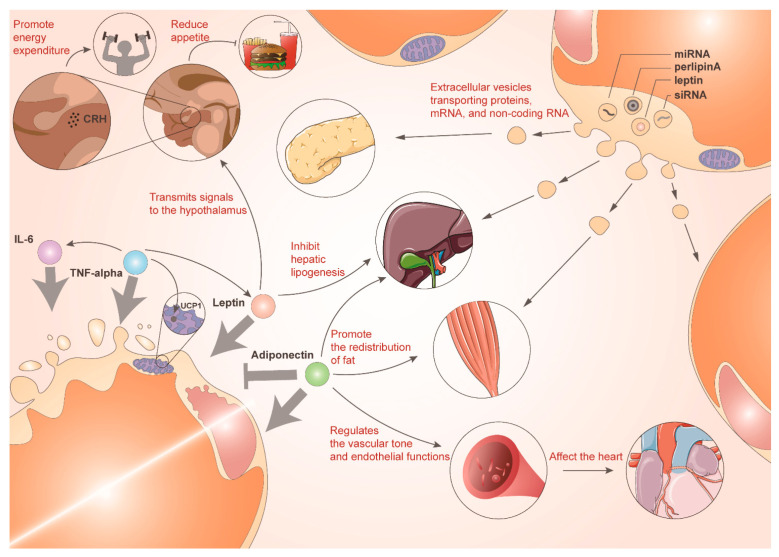
Plasticity of adipocytes secreting adipokines or extracellular vesicles. Adipocytes secrete a variety of adipokines, including adiponectin, leptin, IL-6, and TNF-α, which regulate adipogenesis and lipolysis (lower left panel). The expression of pro-inflammatory cytokines such as IL-6 and TNF-α is significantly reduced under cold conditions. In obesity, the expression of leptin and pro-inflammatory cytokines increases, while the expression of anti-inflammatory cytokines, such as adiponectin, decreases. Leptin inhibits the onset of obesity by establishing a link with the hypothalamus to reduce appetite and promote energy expenditure. Adiponectin releases the adipocyte starvation signal so as to promote the redistribution of fat deposited ectopically in the liver/muscle to the subcutaneous fat depots. In addition, adiponectin regulates the vascular tone and endothelial functions, including coronary arteries, which affect the heart. Adipocytes release extracellular vesicles, transporting proteins, mRNA, and non-coding RNA (e.g., miRNA) to target the organs and cells (upper right panel). In a population affected by obesity, the circulating levels of EVs are significantly elevated, thereby regulating a range of processes, such as inflammation, fibrosis, and tumor progression.

Adipocytes possess important endocrine functions and can secrete various signaling molecules known as adipokines (Figure 4). Adipokines play crucial roles in regulating metabolism, inflammation, and insulin sensitivity, as well as influencing processes such as appetite control and cardiovascular health. Leptin, the first adipokine to be demonstrated, is a critical feedback signal from the brain regarding AT size and status, and it regulates energy expenditure [155]. A deficiency of leptin and its receptors causes severe obesity in relevant mouse models and humans [156]. Leptin primarily transmits nutritional feeding signals to the hypothalamus, resisting obesity onset by reducing appetite and promoting energy expenditure [157]. Leptin also plays a regulatory role, reducing adipogenesis as well as promoting lipolysis, in relation to sympathetic innervation around AT [158]. The expression of the pro-inflammatory adipokines TNF-α and IL-6 is significantly reduced during cold exposure [159]. In obesity, pro-inflammatory pathways within adipocytes, including JNK and NF-κB pathways, are activated and adipokine secretion is altered accordingly. Levels of pro-inflammatory cytokines such as TNF-α [160] and MCP-1 [16] are elevated, whereas those of anti-inflammatory cytokines such as adiponectin are declined [161]. Resistin [162] and retinol-binding protein 4 [163] are also well-studied adipokines.

AT can regulate systemic homeostasis by releasing extracellular vesicles (EVs) that act on other tissues to regulate lipid and glucose homeostasis (Figure 4). EVs are crucial mediators of cellular communication between AT and other tissues. They deliver proteins, mRNAs, and non-coding RNAs to recipient cells, thereby altering their phenotype [164]. Leptin and adiponectin are also expressed in EVs secreted by 3T3-L1 adipocytes and the human SAT [165]. Obesity significantly increases the circulating levels of EVs enriched with perilipin A, an adipose-specific protein useful for detecting circulating adipocyte-derived EVs [165]. miRNAs contained within secreted EVs are altered in obese AT compared with lean AT. miR-148b and miR-4269 are downregulated, whereas miR-23b and miR-4429 are upregulated [166]. The mRNAs predicted by differentially expressed miRNAs are also expected to change with obesity development through the TGF-β and Wnt/β-catenin signaling pathways, thereby regulating inflammation and fibrosis in other organs [166]. Some EVs are taken up by immune cells and can activate pro-inflammatory macrophages that drive local and systemic inflammation and IR [167]. siRNA- and miRNA-containing paracrine EVs secreted by ASCs in the adipose tissue microenvironment of patients with ovarian cancer contribute to tumor progression [168]. The presence of various adipokines and EVs highlights that AT is indispensable in the regulation of the systemic endocrine system.

## 10. Thresholds of Adipose Tissue Plasticity and Their Individualized Differences

AT plasticity in all aspects is not never-ending. Beyond a certain threshold, irreversible damage may occur. In the group with obesity, for example, when AT plasticity is within the threshold, the adipocytes become moderately larger and more numerous, inflammation occurs mildly, angiogenesis increases, and thus hypoxia occurs rarely. This may explain why some infants and adolescents with obesity and people with metabolic health obesity (MHO) do not develop metabolic disorders [169,170,171]. By contrast, when AT plasticity exceeds the threshold, in terms of adipocyte size regulation, FFA leaks into the cytoplasm as lipid droplets reach their maximum storage limit. This increases oxidation and endoplasmic reticulum stress in adipocytes, which can result in cellular dysfunction, inflammation, and ultimately contribute to metabolic disorders [172]. SAT is the largest and best storage site for excess lipids. However, its limited ability to recruit precursor cells reduces the production of new adipocytes, forcing reliance on hypertrophy for lipid storage, which ultimately leads to unhealthy AT expansion accompanied by increased inflammation and IR [95,173]. Regarding adipose ECM remodeling, excessive infiltrating macrophages impair mature adipocyte generation, thereby disrupting ECM homeostasis, resulting in fibrotic deposition, and exacerbating inflammation [174]. Limited angiogenesis and subsequent hypoxia are also characteristics of pathological adipogenesis [175]. When the threshold of AT plasticity is breached, hypoxia, fibrosis, inflammation, and other adverse effects occur back-to-back and interact to disrupt body homeostasis.

**Figure 5:**
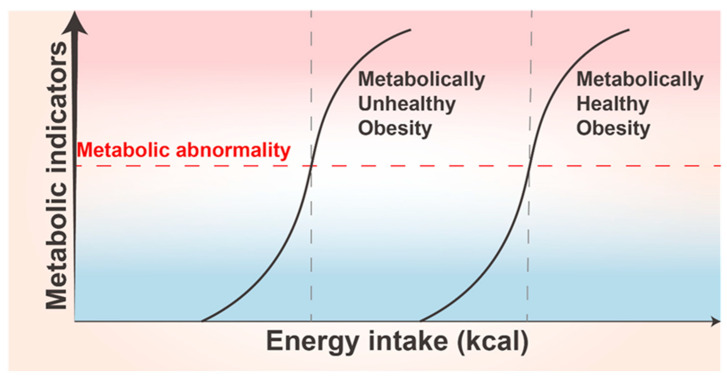
Individual differences in adipose tissue plasticity. Metabolically unhealthy obese (MUO) populations demonstrate a lower threshold for adipose tissue plasticity compared to metabolically healthy obese (MHO) populations, where the latter exhibit a higher threshold. In cases of equal energy intake, MUO individuals are more susceptible to metabolic disorders, characterized by abnormal increases in various metabolite types and densities. These aberrations subsequently lead to disturbances in a range of metabolic indicators.

AT plasticity thresholds vary for individuals. Significant differences exist between individuals in response to excessive energy intake, which may explain the presence of MHO and metabolically unhealthy obesity (MUO) in clinical practice [176] (Figure 5). The physiological basis for MHO existence may be the high threshold of AT plasticity in a population subset. Despite their potential for metabolic abnormalities, because of high threshold of AT plasticity in MHO, the body has a higher capacity for adaptive changes and thus does not show metabolic abnormalities. Genetic variants are an essential influence. Genome-wide association studies have identified a set of loci-harboring genes that possibly control excess body fat distribution and metabolism, with 14 genetic variants associated with a lower metabolic abnormality risk. A study associated with UK Biobank revealed that 11 of these variants were related to a higher body mass index (BMI) and adiposity. This implies that some individuals with obesity are possibly at a lower risk of metabolic abnormalities, partially explaining the reason for the presence of the MHO phenotype [177]. Age differences also contribute to individual differences in AT plasticity. The BAT thermogenic activity progressively decreases with aging, with reduced metabolic capacity and greater susceptibility to chronic diseases [131]. The ASC differentiation and proliferation potential decrease considerably with age, and so does the ability of preadipocytes to differentiate and store lipids, allowing more lipotoxic FFAs to harm other organs [178].

## 11. Relationship between Adipose Tissue Plasticity and Disease

AT plasticity is an active adaptive capacity of AT to self-regulate in response to various bodily changes and to protect the body to a certain degree. As mentioned above, this protective capacity is limited, and beyond the limit, it can contribute to the development of various diseases and turn into an injury.

### 11.1. Obesity

A hallmark of obesity is unhealthy AT expansion, including excessive hypertrophy and hyperplasia of adipocytes, often beyond the plasticity threshold in adipocyte size and number. Dysfunctional AT also exhibits varying degrees of inflammation, impaired energy metabolism, and cellular senescence, while inducing several systemic complications by promoting ectopic lipid overflow and the release of different adipokines [1]. Persistent inflammation in AT contributes significantly to obesity development. Compared with WAT, BAT from HFD-fed mice exhibits lower immune cell enrichment, inflammatory marker expression, and macrophage infiltration, suggesting that BAT can resist obesity-induced inflammation [179]. Despite some anti-inflammatory mechanisms of BAT, a sustained and intense local inflammatory environment and some pro-inflammatory cytokines directly impair the thermogenic and metabolic activities of BAT and beige AT. This severely affects energy expenditure mechanisms and leads to obesity-related metabolic syndrome and a series of cardiovascular alterations [16,180]. BAT activation and the induction of WAT browning accelerate glucose and lipid uptake and reduce insulin secretion requirements. This may be a novel strategy for improving glucose and lipid metabolism and IR in people with obesity and those with T2DM [86]. The inflammatory environment of AT also disrupts ECM homeostasis, leading to fibrotic deposition. Fibrosis further limits AT healthy expansion in terms of both physical restriction and functional impairment, exacerbating obesity progression [181,182]. The plasticity of adipogenesis is also impaired in obesity development. The preadipocyte proportion in the SAT is reduced in obesity, with a corresponding reduction in the generation of new healthy adipocytes promoting AT dysfunction [183]. Pro-inflammatory macrophages also disrupt de novo adipogenesis in WAT by secreting pro-inflammatory cytokines that inhibit preadipocyte differentiation and impair normal signaling pathways, such as the insulin and PPARγ pathways, leading to increased lipotoxicity and metabolic dysfunction [174].

### 11.2. Lipodystrophy

Lipodystrophy is a rare disease characterized by selective AT loss. It often manifests as a decrease in SAT of the trunk and extremities and an increase in VAT and fat of the head and neck [184]. Very little amounts of AT can have the same adverse effect on the body’s metabolism as very large amounts of AT. Similar to that in people with obesity, excess lipids are transferred to the liver, bones, and other tissues for ectopic deposition in patients with lipodystrophy and are associated with IR development [185]. Such IR can be improved by the leptin’s action [186]. In a patient with autoimmune lipodystrophy, P. Fischer-Posovszky et al. found that AT loss was associated with cellular inflammation in lymphoid tissue and increased TNF-α concentrations. TNF-α and interferon-γ stimulated CD95 expression and enhanced the formation of CD95 death-inducing signaling complexes in adipocytes, suggesting that CD95-induced apoptosis mediates adipocyte loss [187]. The endoribonuclease Dicer regulates the plasticity of AT species. In HIV-infected patients with lipodystrophy, reduced Dicer levels lead to increased BAT whitening and the downregulation of BAT and beige AT-related gene expression, which triggers metabolic dysregulation [188].

### 11.3. Insulin Resistance

IR is a pathological state often correlated with obesity and the development of various obesity-related complications. IR development is also inextricably linked to AT plasticity. Hypertrophic adipocytes activate the JNK and NF-κB pathways, initiating the transcription of pro-inflammatory genes and the secretion of adipokines such as TNF-α and IL-6. This process triggers localized and systemic chronic inflammation, which impairs insulin signaling pathways, leading to insulin resistance. Furthermore, macrophage infiltration is promoted, exacerbating the development of T2DM [189]. Lipodystrophy and obesity, as previously described, lead to excessive lipid deposition in other organs such as the liver and muscle, which are hallmarks of and major contributors to IR [190]. Lactate links IR and obesity. Slc16a1, which encodes the major lactate transporter protein, when deleted in adipocytes, can lead to intracellular lactate accumulation. This induces apoptosis and the release of large amounts of pro-inflammatory adipokines, thereby causing persistent inflammation and systemic IR [191]. EVs are critical tools in IR progression, and in addition to the previously described EV functions, EVs may contribute to the exacerbation of cognitive impairment and synaptic damage by IR [192]. Macrophage-produced peptidase D promotes AT fibrosis, while excessive AT fibrosis exacerbates IR progression [193]. New-onset adipogenesis promotes healthy AT expansion and can prevent obesity-associated IR ([194], p. 2). While high lipolysis rates are also associated with IR, serotonin receptor 2B (HTR2B)/5-hydroxytryptamine signaling in AT may be involved in lipolysis regulation. HTR2B expression levels are elevated within VAT in people with obesity, and selective HTR2B antagonists may attenuate the inflammatory response in VAT and obesity-associated IR [195]. L. Wang et al. identified a new cell line, p21^Cip1^ (Cyclin-dependent kinase Interacting Protein 1)-highly expressing (p21^high^) cells, through single-cell transcriptomics. These cells in AT cause IR in vivo, which is possibly associated with the NF-κB pathway. Intermittent clearance of p21^high^ cells can alleviate IR [196].

### 11.4. Polycystic Ovary Syndrome

PCOS is a common endocrine disorder observed in premenopausal women. It is characterized by hyperandrogenism and ovarian dysfunction-related symptoms. PCOS is affected by various epigenetic and environmental factors [197]. High androgen levels can promote the differentiation from preadipocytes to mature adipocytes in the abdomen, and thus aggravate the visceral obesity phenotype [198]. AT regulates PCOS courses mainly through adipokine release. Elevated levels of AT-secreted leptin in women with obesity suppress insulin-induced ovarian steroid production and inhibit the pro-estradiol production of the luteinizing hormone (LH) [199]. Serum adiponectin levels increase with weight loss and have a regulatory influence on the central reproductive endocrine axis, such as inhibiting the release of LH and the gonadotropin-releasing hormone [200]. Decreased levels of endometrial adiponectin receptors AdipoR1 and AdipoR2 are correlated with implantation failure during pregnancy in women with PCOS [201]. In a randomized controlled study, serum resistin levels were significantly lower in people affected by obesity and PCOS who were treated with the insulin sensitizer rosiglitazone, implicating its potential utility in PCOS treatment [202]. The levels of pro-inflammatory adipokines such as MCP-1 were also significantly elevated in patients with PCOS, whereas TNF-α and IL-6 levels were not altered [203]. Ovarian dysfunction and hyperinsulinemia in patients with PCOS are strongly associated with the persistent and chronic inflammation of AT, and both obesity and IR contribute to PCOS progression by exacerbating inflammation [204].

### 11.5. HAIR-AN Syndrome

HAIR-AN syndrome is characterized by hyperandrogenemia (HA), IR, and acanthosis nigricans (ANs) and is mainly caused by AT dysfunction [205]. A cross-sectional study found that patients with HAIR-AN syndrome had higher leptin levels and lower adiponectin levels than those with PCOS, which implies a more significant role of adipokines in HAIR-AN syndrome [206]. IR and HA have been described previously, where AN was skin pigmentation and hyperkeratosis exhibited in the axillae, posterior neck, etc. [207]. This cutaneous manifestation may predict hyperinsulinemia in people with obesity but without diabetes [208]. Symptoms of AN have also been reported in patients with acquired lipodystrophy and hereditary IR syndrome [209,210].

### 11.6. Atherosclerosis

A key contributor to atherosclerosis is obesity-induced adipose dysfunction, and ectopic fat deposits, such as in the liver, and epicardial fat deposits also increase the atherosclerosis risk [211]. Adipocyte hypoxia-inducible factor 2α may exhibit anti-atherosclerotic effects by reducing hepatic cholesterol levels [212]. The AT distributed around blood vessels, called perivascular AT (PVAT), is a critical factor affecting cardiovascular system homeostasis, and a bidirectional regulatory pathway exists between the vessel wall and PVAT [213]. PVAT is as malleable as AT in other locations. Physiologically, PVAT provides mechanical support and temperature maintenance for the vasculature. Moreover, it regulates vascular tone and endothelial function by secreting adipokines such as adiponectin [214]. Adiponectin synthesis is regulated by circulating BNPs [214]. However, during vascular injury, PVAT undergoes inflammatory changes and long-term inflammation can lead to pathological vascular remodeling [215]. BMP4 may inhibit atherosclerosis progression by promoting the browning of PVAT, which increases mitochondrial activity and thermogenesis, and by exerting anti-inflammatory effects through the reduction in pro-inflammatory cytokines and macrophage infiltration; browning is considered a protective mechanism of PVAT [216].

### 11.7. Metabolic-Associated Fatty Liver Disease

MAFLD, also previously known as non-alcoholic fatty liver disease, is closely related to metabolic diseases such as obesity. It increases the danger of liver failure and hepatocellular carcinoma [217]. In fatty liver disease, the lipid content of the liver increases and adipocytes are ectopically deposited in the liver, which is the outcome of a change in the plasticity of AT distribution. Macrophages have a critical role in MAFLD development, and dysregulated AT metabolism can activate hepatic macrophages in MAFLD [218]. Pro-inflammatory macrophages are correlated with MAFLD severity [219]. Adiponectin promotes macrophage polarization to an anti-inflammatory phenotype. Experiments have demonstrated that adiponectin KO mice are more susceptible to steatohepatitis, whereas adiponectin administration delays steatohepatitis progression [220]. Leptin levels were also elevated in patients with MAFLD and may mediate liver fibrosis by affecting Kupffer cells [221]. AT inflammation may occur earlier than liver tissue inflammation in MAFLD pathogenesis [222]. PPAR-γ agonists exert an ameliorative effect on hepatic steatosis in patients with MAFLD by inducing the anti-inflammatory phenotype of macrophages [223].

### 11.8. Tumor

AT plays a crucial role in the development and progression of various tumors. Adipocytes facilitate tumor metastasis, provide energy for rapid tumor growth, and produce signaling molecules that stimulate cell invasion and migration [224]. In ovarian cancer, human omental adipocytes promote cancer cell homing, migration and invasion by secreting adipokines such as IL-8 [225]. In breast cancer, FFAs generated from adipocyte lipolysis are transferred to cancer cells, fueling their fatty acid metabolism and enhancing tumor growth [226]. Similarly, in melanoma, adipocyte-derived lipids contribute to tumor progression through fatty acid transporter protein [227]. Moreover, obesity-associated alterations in inflammation and adipokine signaling, including elevated leptin levels and reduced adiponectin, further promote tumor growth and metastasis [228].

## 12. Mechanisms and Drugs Targeting Adipose Tissue Plasticity in Obesity Treatment

**Figure 6:**
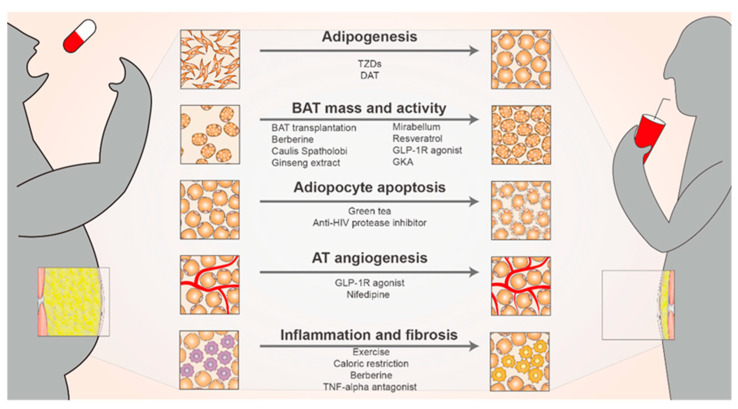
Mechanisms and drugs targeting adipose tissue plasticity in obesity treatment. Mechanisms including the regulation of adipocyte production and AT expansion increase in the BAT mass and activity, regulation of adipocyte apoptosis, improvement in adipose tissue angiogenesis, and improvement in inflammation and fibrosis in the adipose tissues. The drugs associated with each of the therapeutic mechanisms are listed. AT: adipose tissue. BAT: brown adipose tissue. TZD: thiazolidinediones. DAT: decellularized adipose tissues. GKA: glucokinase agonists.

During obesity, to accommodate excess energy, AT undergoes diverse changes, including adipocyte hypertrophy, hyperplasia, and remodeling of the vascular system and ECM. This allows sufficient AT expansion to coordinate the mobilization of various nutrients [229]. However, after a certain limit is exceeded, several hazards such as persistent inflammation, fibrosis, and hypoxia ensue. The adverse effects of obesity originate in AT pathological remodeling [175]. Many treatments have been reported for various aspects of adipose plasticity (Figure 6).

### 12.1. Regulation of Adipocyte Production and Adipose Tissue Expansion

The failure of adipose precursor cells to form new adipocytes is among the major causes of AT dysfunction and is also closely related to obesity development [1]. PPARγ is a primary regulator of adipogenesis, and the PPARγ agonist TZD drives sirtuin 1-dependent PPARγ deacetylation, facilitating the interaction of PPARγ with PRDM16 and inducing metabolic and cellular remodeling. This thus improves AT’s ability to sequester fatty acids [230,231,232]. Decellularized adipose tissue (DAT) does not contain cellular structures but is rich in collagen, which thus creates a good microenvironment for adipogenesis. DAT is considered an ideal derived scaffold for AT by interacting with different types of MSCs for eventual adipose regeneration [233]. Enabling massive SAT expansion has been shown to counteract excessive caloric intake-associated IR and to improve lipid metabolism [132].

### 12.2. Increase in BAT Mass and Activity

BAT atrophy and increased visceral fat are observed in people with obesity; therefore, increasing BAT mass or activity is considered a potential strategy for obesity treatment [234]. BAT transplantation significantly prevents weight gain, reduces total fat weight, and increases oxygen consumption in mice with obesity, hence improving IR in them [235], which may be partially mediated by IL-6 [236]. Berberine [237], the water extract of Caulis Spatholobi [238], and ginseng extract [239] can improve obesity through BAT activation. The latter two can induce an increase in anti-obesity-associated bacteria by modulating gut microbiota composition. Although β3-AR agonists such as mirabellum can activate BAT, they have cardiovascular side effects and their use should be considered with caution [240]. Activated BAT can prevent obesity and T2DM by absorbing and utilizing glucose and lipids, improving IR, and reducing blood glucose, lipids, and total fat mass [241].

The molecules and regulatory pathways associated with WAT browning are new targets for obesity treatment [242]. Resveratrol, a natural antiaging polyphenol, increases the activation of the SIRT1 signaling pathway in AT to promote WAT browning [243] and is correlated with adipocyte apoptosis [244]. The GLP-1 receptor agonist liraglutide increases the expression of BAT marker genes in the SAT of diabetic rats, promotes SAT browning, and reduces VAT weight [245]. Glucokinase, an emerging antidiabetic target, and glucokinase agonists (GKA), such as the newly marketed dorzagliatin, can be used in routine diabetes treatment. GKA is safe, promotes insulin and GLP-1 secretion, and has potential weight loss effects [246]. The central browning agent nicotine may act through the κ opioid receptor in the lateral hypothalamus [247]. Retinoic acid [248], menthol [249], allicin [250], and pentamethylquercetin [251] have been found to promote WAT browning while exhibiting efficacy in reducing body weight and improving insulin sensitivity. However, differences in the browning capacity of individual fat depots may limit the therapeutic value of browning agents; moreover, VAT is less susceptible to browning agents than SAT [240].

Brown-like adipocytes, designed by CRISPR-Cas9 technology, and tested in mice for obesity and metabolic syndrome treatment have exhibited good efficacy and may offer therapeutic opportunities at the cellular level. Specifically, human brown-like (HUMBLE) cells were created by modifying human white preadipocytes with CRISPR/Cas9-SAM-gRNA to activate UCP-1 expression. The transplantation of HUMBLE cells into obese mice resulted in sustained improvements in glucose tolerance, insulin sensitivity, and increased energy expenditure [252].

### 12.3. Regulation of Adipocyte Apoptosis

Reducing adipocyte number by regulating adipocyte apoptosis can be complementary to other therapeutic options for obesity [253]. Green tea activates apoptotic signaling in adipocytes, with its active component possibly being (−)-epigallocatechin-3-gallate (EGCG). A reduction in the mass of individual fat depots was observed in EGCG-fed mice [254]. Lipodystrophy has been described in the treatment of patients with AIDS using the anti-HIV protease inhibitor ritonavir. This may be related to the apoptosis-inducing effect of ritonavir [255].

### 12.4. Improvement of Adipose Tissue Angiogenesis

Promoting angiogenesis can effectively improve local hypoxia, fibrosis, and inflammation caused by rapid AT expansion, but excessive angiogenesis may lead to unhealthy WAT expansion [256]. Therefore, whether angiogenesis inhibitors or promoters should be chosen to treat obesity remains controversial. Exenatide, a GLP-1 receptor agonist, increases AT angiogenesis through VEGF A in people with obesity and diabetes, improves AT chronic hypoxia, and alleviates obesity and obesity-related metabolic disorders [257]. Nifedipine, an antihypertensive drug, can improve obesity-impaired angiogenesis by inhibiting oxidative stress and increasing the number of endothelial progenitor cells. This thus improves AT hypoxia, increases mitochondrial thermogenesis, and halts obesity development [258]. Metformin has been found to have a role in obesity treatment. However, the exact mechanism remains unclear, and the inhibition of AT angiogenic activity is among the possible mechanisms [259].

### 12.5. Improvement of Inflammation in Adipose Tissue

Obesity promotes an inflammatory environment within the AT and exacerbates fibrosis. Furthermore, obesity alters ASC composition and function. Applying good lifestyle modifications, such as exercise and caloric restriction, may suppress or even reverse this class of pathological changes [260]. The regulation of different inflammation-associated pathways in macrophage polarization, thereby inhibiting M1 polarization and reducing inflammatory factor release, may be a potential strategy for obesity treatment [108]. By inhibiting M1 macrophage activation in AT, berberine may improve IR induced by chronic inflammation in AT, which in turn reduces serum TNF-α and IL-6 levels [261]. The TNF-α antagonist etanercept used for the treatment of obesity and related metabolic complications may alleviate obesity-induced inflammation and reduce fasting glucose levels by modulating adiponectin levels. Nevertheless, the wide application of etanercept is limited because of its possible side effects such as increased susceptibility to infection [262].

## 13. Discussion

Obesity is a highly prevalent and harmful condition and is associated with a wide range of complications. Obesity is ultimately caused by abnormal fat metabolism, and altered AT plasticity is a sign of abnormal fat metabolism. We here first provide the complete definition of AT plasticity. At the same time, we emphasize that a threshold of AT plasticity exists, which may be related to the presence of MHO and MUO. This AT plasticity allows the body to adapt rapidly to various alterations in energy requirements, and the underlying process is regulated by multiple factors [263].

AT expansion manifests itself as adipocyte enlargement or an increase in adipocyte number, which begins in fetal life and continues throughout the life cycle [264]. Changes in AT species are an essential part of AT plasticity, and far more studies have investigated the AT browning process than the whitening process. In addition to traditional fat depots, a natural class of beige adipose depots, called thigh adipose tissue (tAT), was recently identified and characterized in mice maintaining a beige adipose morphology at room temperature. tAT exhibits significantly higher BAT marker expression and a considerably greater browning potential than iWAT, and its UCP1 content is 2600-fold higher than that of iWAT under HFD stimuli, which may be a more suitable model for studying AT plasticity [265].

A unique type of AT known as “pink adipocytes” received its name due to its pink color during pregnancy and lactation. These cells are alveolar in nature and contain large cytoplasmic lipid droplets. In appearance, they closely resemble adipocytes and can transdifferentiate from WAT. A new concept, termed “pinking,” refers to the process of white-to-pink transdifferentiation or adipoepithelial conversion [266]. Perilipin1 is expressed in white adipocytes but not in pink adipocytes, while perilipin2 is characteristic of pink adipocytes and is absent in white adipocytes [267]. Lineage-tracing experiments have also confirmed the transdifferentiation of WAT into pink adipocytes [266]. During lactation, lipids are transferred from adipocytes to alveolar cells, which secrete fat as a component of milk. After lactation, the cytoplasm of lean adipocytes is refilled with lipids, allowing them to regain the typical WAT morphology [85]. Cells with intermediate features of pink and brown adipocytes have been identified in the dorsal mammary glands of post-lactation mice, where BAT in the interscapular region is located near the alveoli. These cells display several ultrastructural markers of brown adipocytes, such as multilocular lipid droplets and elevated UCP1 expression, indicating the transition from pink to brown adipocytes [268,269].

MSCs from the mesoderm are now widely considered a source of APCs [270,271]. Adipose single-cell mapping has confirmed the complex APC heterogeneity in mouse and human adipose depots [2]. APC profiling has revealed that most brown adipocytes originate from myogenic Myf5+ MSCs, whereas white adipocytes from adipogenic Myf5− MSCs. Indeed, both brown and white adipocytes originate from multiple lineages, which is possibly related to their plasticity [272]. The regulatory factors involved in preadipocyte differentiation and adipocyte dedifferentiation have been mentioned previously. Obesity induces both structural and functional changes in mitochondria within human MSCs, including decreases in mitochondrial matrix density and fatty acid metabolite levels [273]. Adipogenic regulatory cells (Aregs), which are a type of preadipocyte, were recently identified in mice and humans. Aregs are characterized by elevated Cd142 and Abcg1 expression and exert antiadipogenic effects on committed progenitor cells through a paracrine mechanism [274].

The threshold of AT plasticity simply means that the degree of AT changeability is limited by many aspects. The plasticity of AT distribution may be of wide interest due to its lipid-lowering effects. Collagen and proteoglycans are crucial ECM components in AT. They are present at an appropriate density in the healthy state, while their excessive accumulation leads to severe fibrosis, thereby promoting AT dysfunction [275]. AT over-expansion and the inability of angiogenesis to meet its demands lead to hypoxia and senescent cell accumulation, aggravating AT fibrosis and inflammation, and uncontrolled lipid spill over, producing lipotoxicity [92]. The cause–effect relationship is complex, and identifying the initiating factor and the outcome is difficult. The relationship between the presence of MUO and MHO and AT plasticity needs to be further explored clinically.

## 14. Prospect

AT has been a focus area of energy metabolism research for a long time, and recent advancements, such as scRNA-seq and spatial transcriptomics, have deepened our understanding of complex AT dynamics [260,276]. The WAT-on-Chip system has created the first human-based, autologous, and immunocompetent AT model in vitro. This system uses organ-on-chip technology to almost reproduce WAT heterogeneity and is expected to be a powerful tool in metabolism and related fields [277]. AT plasticity research will become a hot spot in the future, and more drugs related to AT plasticity regulation will be invented and developed. Therapies related to ADSC regulation and differentiation will receive attention because of their abundant source and high self-renewal potential. AT plasticity, as an active changeability, is irreplaceable in disease development. The multidimensional dissection of AT plasticity facilitates an in-depth understanding of adaptive changes in AT in response to systemic physiological or pathological changes and provides multiple potential targets for obesity treatment strategies to be applied in clinical practice.

## 15. Conclusions 

In conclusion, AT plasticity is a complex and adaptive process that plays a central role in growth, obesity, organismal protection, and homeostasis. This plasticity involves dynamic changes in adipocyte size, number, lipid storage, and cellular composition, all of which are crucial for the tissue’s response to physiological and pathological stresses. Understanding the mechanisms behind AT remodeling offers valuable insights into its contributions to various diseases. By exploring AT plasticity, we can identify new potential therapeutic targets for managing obesity and other metabolic disorders, ultimately advancing relevant clinical applications.
